# The Digestive Health among Participants of the Woodstock Rock Festival in Poland—A Cross-Sectional Survey

**DOI:** 10.3390/ijerph15102256

**Published:** 2018-10-15

**Authors:** Karolina Skonieczna-Żydecka, Ewa Stachowska, Dominika Maciejewska, Karina Ryterska, Joanna Palma, Maja Czerwińska-Rogowska, Mariusz Kaczmarczyk, Anna Gudan, Honorata Mruk, Barbara Świniarska, Justyna Kałduńska, Zofia Stachowska, Przemysław Mijal, Tomasz Mazur, Maciej Kupczyński, Wojciech Marlicz

**Affiliations:** 1 Department of Biochemistry and Human Nutrition, Pomeranian Medical University, 71-460 Szczecin, Poland; karzyd@pum.edu.pl (K.S.-Ż.); dmaciejewska.pum@gmail.com (D.M.); ryterska.karina@gmail.com (K.R.); palma.01.01@gmail.com (J.P.); majaczerwinska89@gmail.com (M.C.-R.); gudananna@gmail.com (A.G.); honorata.m@smsnet.pl (H.M.); swiniarskab@gmail.com (B.Ś.); justynakaldunska@wp.pl (J.K.); zofia.stachowska.1994@gmail.com (Z.S.); Przemyslaw.mijal@gmail.com (P.M.); tomasz.mazur90@gmail.com (T.M.); maciejkupczynsky@gmail.com (M.K.); 2 Department of Clinical and Molecular Biochemistry, Pomeranian Medical University, 70-111 Szczecin, Poland; mariush@pum.edu.pl; 3 Department of Gastroenterology, Pomeranian Medical University, 71-252 Szczecin, Poland; marlicz@hotmail.com

**Keywords:** microbiota, digestive health, defecation, stress, functional gastrointestinal disorders, FGIDs

## Abstract

Alterations of gut microbiota, intestinal barrier and the gut-brain axis may be involved in pathophysiology of functional gastrointestinal disorders. Our aim was to assess the prevalence of digestive tract symptoms and identify common variables potentially disrupting the gut-brain axis among participants of the Woodstock Festival Poland, 2017. In total 428 people filled in a questionnaire assessing health of their digestive tract. The investigator collected answers on an electronic device, while the study participant responded using a paper version of the same questionnaire. Liver and gallbladder related symptoms were the most prevalent among our study group (*n* = 266, 62%), however symptoms related to altered intestinal permeability were found to be the most intensive complaints. In females the intensity of gastrointestinal complaints was higher compared to men (*p* < 0.05), as well as the incidence of factors with the potential to alter gut-brain axis (*p* < 0.0001). Chronic psychological distress, intake of non-steroidal anti-inflammatory drugs (NSAIDs) and antibiotics, were the most common associations with gastrointestinal symptoms, which were the most prevalent in females. Further attention should be focused on stress as one of the main factors negatively influencing public health.

## 1. Introduction

Functional gastrointestinal disorders (FGIDs) are common in the general population, with a prevalence of 10–30% depending on the methodology but also on gender and ethnicity [1,2]. In pediatric populations it was estimated that about 25% of children under the age of 18 years fulfilled various criteria for FGIDs [3].

FGIDs frequently overlap alimentary tract symptoms described until recently as unrelated to any physiological mechanisms [4]. However, the scientific data is mounting that the phenotype of the disease is an exponent of gut-brain interaction, mediated by visceral hypersensitivity, motility alterations, skewed immune and mucosal function and dysbiosis [5,6]. Despite that, symptomatological significance is not well established, which introduce challenges and uncertainty into clinical practice [1].

With the advances in culture-independent biotechnologies it was shown that human digestive tract microbiota are made up of trillions of microbials, forming heterogenous, multispecies and interactive communities [7]. In human fecal samples, researchers identified almost 10 billion microbial genes [8]. Human bacterial microbiota are thought to be quite stable from childhood until the old age, however on strain level may vary significantly among persons [9]. Overall, the variations within microbiomes are rather consistent in healthy populations [10,11]. Microbiota was identified as critical for nutrient breakdown and xenobiotic metabolism [12,13], protection against pathogens and epithelial injury [14,15], immunomodulation [16,17], vascularization [18], modulation of bone-mass density [19], fat storage [20], vitamin synthesis [21] and modification of the nervous system [22]. However, microbiota is not an isolated organ. Microbiota in combination with the gut associated-lymphoid tissue (GALT), epithelial monolayer, as well as the circulatory, lymphatic and nervous system co-create the intestinal barrier separating luminal and internal environments [23]. It wasproven that intestinal barrier disruptions, encompassing dysbiosis and consequently tight junctions structure alterations lead to GALT activation. It is of particular relevance, as FGIDs may be developed secondary to acute GI infections, resulting for example in post infectious irritable bowel syndrome (PI-IBS). A few studies found elevated numbers of GALT effector cells as well as biochemical mediators within intestinal barrier of FGIDs patients but without overt clinical manifestation [24,25,26]. Literature has also proved that immune activation is accompanied by sensory and motor dysfunctions within the gut [27]. Enteric nervous system (ENS) passes agitation using, among others, rapid acting catecholamines and slow acting neuropeptides like nitric oxide (NO) and calcitonin gene related peptide (CGRP). It was proved that inflammatory response is partly regulated by sensory neuron actions via elevating the production of neuropeptides and producing neurogenic inflammation by means of vasodilatation and plasma extravasation. For example, the suppression of endogenous NO suppressed the blood flow in stomach [28] and elevated the intensity of pancreatic damage [29] while the presence of CGRP aggravated pancreatic damage [30], also in the case of the caerulein-induced pancreatitis animal model [31]. On the other hand, it was shown that the presence of norepinephrine has a negative effect on bacteria and viruses, increasing their pathogenicity, including the ability to adhere to intestinal epithelial cells. This, in turn, induces an increase in the pathogen capture by dendritic cells, which in turn leads to a massive presentation of antigens to lymphocytes and a further increase in the intensity of inflammation [32]. To close the circle, different gut microbiota metabolites regulate the function of the myenteric plexus, thus affect visceral perception, motility, as well as secretory and motor functions of the GI tract [33,34,35,36]. For example, short chain fatty-acids being by-products of gut microbiota stimulate colonic blood flow and gut motility [37]. Also, environmental stimuli, including psychological stress were recognized as gut barrier integrity disruptors [38]. Lastly, Stassi et al. [39] analyzed psychopathological dimensions in FGIDs patients and found that at least a single psychiatric disorder, predominantly of depressive spectrum, was present in almost half of the patients enrolled. These all underlie that FGIDs are sourced from gut-brain axis dysfunction. All in all, there has been huge progress in the field of microbiome-gut-brain axis with the focus on intestinal barrier integrity, which have become attractive targets for prophylaxis and treatment of common FGIDs.

The data on FGIDs prevalence in Poland are scarce and large-scale investigations in Poland are lacking. As reported by Niemyjska et al. [40] the frequency of abdominal pain occurring at least once every week in a cohort of Warsaw University was found to be around 50% and it varied in terms of psychological and physical stress experience. Ziółkowski et al. [41], found that the frequency of dyspepsia, constipation and bloating in a middle-size polish city is around 23%, 13% and 31% respectively.

Our preliminary report [42] proved that the frequency of abdominal pain—being a major ROME IV diagnostic criterion of IBS—was found to be as high as 19.2% within a sample of approximately 1500 young people in Poland, with psychological distress, proton pump inhibitors (PPIs) and antibiotic usage identified as significant risk factors. We decided to repeat the study using an expanded questionnaire to illustrate the prevalence symptoms of several gastrointestinal tract disorders. We also focused our attention on environmental factors, including nutrition and lifestyle elements, as potential pathophysiological factors of FGIDs.

## 2. Materials and Methods

### 2.1. Sampling and Data Collection

The cross-sectional survey was conducted in Kostrzyn, Poland, during the free admission Rock Woodstock Festival (2–5 August 2017). Festival attendants (predominantly young adults) came from all regions of Poland thus the study may be considered as a representative sample for the country. The data were collected by trained collaborators, both academic scientists and young dietitians. Each of the investigators was provided with an electronic device (tablet/smartphone) in which the questionnaire was installed as an application. Randomly selected participants of the festival were asked to complete a questionnaire on their lifestyle, medication and gastrointestinal complaints. Each of the respondents received a printed version of the questionnaire and passed the answers to the interviewer who marked them to the application. The Medical Ethical Committee of the Pomeranian Medical University in Szczecin evaluated that as questionnaire was anonymous, the respondents would not be contacted again, and their answers would not be stored in foreign internet servers (questionnaire was an application), written informed consent was not obligatory (Ethical Approval Code). However, each eligible study responder volunteered to participate in the experiment and personally pointed it out to the electronic device used in the study. Almost 100% (99.53%) approached persons were included into the study group. We agreed that the inclusion criteria were: Age above 18 years, Polish native speaker and deliberate oral consent to take part in the study. Anyone who failed meeting these criteria were excluded. Also, only completed surveys were analysed. Age and gender of participants were collected.

### 2.2. Questionnaire

The Digestive Health Appraisal Questionnaire (DHAQ) by Lipsky [43] was used in the present study. The survey is divided into two parts. In first part, the study subjects had to specify which of medications and foods they were using. Additionally, questions concerning living under chronic stress, physical activity, and exposure to intoxicants were asked. The number of indications from this part was counted separately (medications, food, lifestyle) and summed up all together to obtain a number expressing the degree of exposure to agents with potential to induce dysbiosis, further called dysbiotic agents or factors. In the second part of the DHAQ, respondents were asked to give the intensity of gastrointestinal symptoms they were experiencing. This self-reported assessment of the digestive health was performed in single-item scale, ranging from 0 (symptoms occur almost never or extremely rare) to 3 (Symptoms are intense/occur almost every day). If a patient did not know the answer it was left blank. The second part of DHAQ was divided into seven sections, corresponding to the following digestive tract symptoms/location:

Section A: Hypoacidity of the Stomach
Section B: Hypofunction of Small Intestines and/or Pancreas
Section C: Ulcers/Hyperacidity of the Stomach
Section D: Colon/Large Intestine
Section E: Liver/Gallbladder
Section F: Intestinal Permeability/Leaky Gut Syndrome, Dysbiosis
Section G: Gastric Reflux

Raw data, i.e., the numbers in each section were summed up to guage intensity of complaints and then expressed qualitatively as recommended by the author of DHAQ as shown in Table 1 below.

### 2.3. Statistical Analyses

The assessment of distribution of continuous variables by the Shapiro-Wilk normality test was done. Nonparametric Mann-Whitney test was used to compare the intensity of digestive tract symptoms in terms of gender and the occurrence of particular dysbiotic agents (medications, permanent distress). When the digestive health determinants were expressed qualitatively we used Chi-squared tests to compare this variable with number of dysbiotic agents declared by the participants and gender, respectively. We correlated the age and the total score for the dysbiotic part of the DHAQ with the intensity of digestive symptoms by means of *Spearman* rank correlation coefficient analyses. The acceptable probability of error for the first type (the significance level of the test) was assumed to be equal to 0.05. All statistical analyses for this study were performed using the StatView computer software version 5.0 (SAS Institute Inc., Cary, NC, USA). To control type I errors, the false discovery rate (FDR) approach was used. The calculations were performed using the p.adjust function of the stats package in R (R Foundation for Statistical Computing, Vienna, Austria, https://cran.r-project.org).

## 3. Results

### 3.1. Study Group

There were 428 persons included into the study group, with a female preponderance (*n* = 239; 55.8%). We identified five persons who reported no gastrointestinal complaints (three men, two women) and four others (two men, two women) who declared not to be exposed to any of the environmental dysbiotic factors. The most prevalent gastrointestinal symptom was found to be a consequence of elevated intestinal permeability (F section of DHAQ), and the least common were symptoms of gastric reflux (G section of DHAQ). The study group characteristics are shown in Table 2.

We found that females were shown to declare more digestive complaints in comparison to men as presented in Table 3. When the intensity of digestive tract complaints were expressed qualitatively similar results were obtained (Table 4).

### 3.2. Dysbiotic Factors

We noticed that lifestyle dysbiotic factors were more intensively experienced by women (Median (Q3–Q1): Females 1.0 (1.0), Males: 1.0 (2.0), *p* < 0.0001) and a statistical tendency toward taking more medications compared to men was found (Median (Q3–Q1): Females = 1.0 (11.0), Males = 0.0 (6.0), *p* = 0.0714). As the literature states that the most common dysbiotic factors include taking antibiotics, nonsteroidal anti-inflammatory drugs and proton pump inhibitors, as well as living under permanent stress, we compared the intensity of gastrointestinal complaints in association with these variables. As presented in Table 5 permanent distress was found to be associated with intensity of all DHAQ indices included into the second part of the questionnaire.

In the next step of our analyses we summed up all dysbiotic indices from the first part of the DHAQ and correlated this variable with the intensity of digestive complaints. We noticed a weak positive correlation between the number of dysbiotics and each section of the second part of the questionnaire (Table 6). We presented DHAQ in a qualitative manner and by means of the Kruskall-Wallis test confirmed that more dysbiotic agents occurred predominantly in patients with high priority gastrointestinal symptoms (Table 7).

## 4. Discussion

Our cross-sectional study was conducted to establish the prevalence of symptom-based bowel disorders in a sample of adult inhabitants of Poland. We found that digestive complaints are common within Polish adults and predominantly correspond to symptoms associated with liver and gallbladder function (*n* = 266; 62.14%). The prevalence of complaints related to the small intestine and colon also exceeded 50% of the study group.

The incidence of FGIDs has been analyzed in literature extensively. In 2002 Drossmann et al., in their technical report prepared for the American Gastroenterological Association, estimated that irritable bowel syndrome (IBS) was affecting up to 15% of the global population [44]. More recently, the worldwide prevalence of IBS was found to be around 12% [45]. The survey conducted among approximately 6000 inhabitants of United States, Canada and United Kingdom found that the incidence of functional dyspepsia (FD) was around 10% [46]. The same researchers concluded that bowel disorders were present in 28.1% of the population sampled, while gastroduodenal, anorectal, esophageal and gallbladder malfunctions were found in 10.6%, 7.4%, 7% and 0.2% of studied sample respectively [47]. Although epidemiological studies reporting global incidence of FGIDs have high heterogeneity [48], the incidence of digestive-related complaints in our survey is much higher. The most reasonable explanation is symptom overlap in the questionnaire we used. Similarly, current ROME IV criteria seem to function irrespective of symptom overlap [49], as investigators may diagnose the same patients with either FD or IBS [50]. Nevertheless, studies evaluating the worldwide prevalence of FGID sufferers using most recent ROME IV criteria, report the drop down of IBS to 5–6%. At least partly, the discrepancies may be also due to ethnicity thus variations in nutritional habits and other psycho-social factors shaping not only symptoms incidence but also their perception and reporting [51].

When analyzing the intensity of digestive complaints we identified that symptoms associated with increased intestinal permeability and dysbiosis seemed to be the most intense ones. The mean number of points describing the intensity of these symptoms were 7.51 ± 5.3 and 4.95 ± 4.47 (*p* < 0.05) in females and males respectively. Overall, the present study found that around 50% of responders fulfilled symptom-based leaky gut syndrome criteria, of which 1.63% (*n* = 7) were of high priority. The intestinal barrier integrity was found to be diminished in FGID patients. Zhong et al. [52] found high copy numbers of *Streptococcus* and low abundance of *Prevotella, Veilonella, Actinomyces, Atopobium* and *Leptotrichia* in FD patients in comparison to controls. Low trans-epithelial electrical resistance (TEER) thus increased paracellular permeability of duodenal biopsies in FD patients was found by Vanheel et al. [24]. Subclinical inflammation in situ was discussed recently by Talley et al. [53]. Similar results were presented in scientific literature evaluating IBS. Dysbacteriosis and even IBS-related bacterial genera were recognized [54,55,56]. Altered tight junction signaling as well as GALT activation in IBS was also proven [57,58,59].

Dysbiosis within the gut ecosystem that alters gut barrier integrity and leads to elevated intestinal permeability is of multifactorial origin. Very recently it was found that host genetics does not play a pivotal role in shaping one’s microbiome. At least 80% microbiota variability is related to environmental factors, among them diet and medicines [60]. In present study we evaluated the exposure to chronic psychological distress, undertaking physical activity, as well as consuming unhealthy food and particular medications. We found that the most common associations with the intensity of gastrointestinal symptom dysbiotics were chronic psychological distress and intake ofNSAIDs.

Psychoneuroimmunological studies proved that the gut brain axis regulates stress response throughout the human body and may be involved in both the pathophysiology and the clinical course of FGIDs [61,62,63,64]. Corticotropin stimulates the release of tumor necrosis factor-α (TNF-03B1) and nerve growth factor contributing to the decrease in tight junction (TJs)protein expression. In addition, the barrier effect has been shown to be dependent on eosinophil-derived proteins. They cause a decrease in occludin content, which further weakens TJs. Stress also intensifies the state of dysbiosis, impairs immune function due to the absence of secretory immunoglobulin A and diminishes regeneration of the mucous membrane. An elevated number of harmful microorganisms damages the intestinal wall and increases their permeability [65,66]. Diet is also critical for microbiota composition and thus intestinal barrier integrity. The consumption of a Western diet high in animal fat and protein was found to decrease the abundance of beneficial *Lactobacillus* and *Bifidobacterium* genera and increase the counts of *Bacteroides* and *Enterobacteria* [67]. A high-fat diet was found to increase bacterial lipopolysaccharide concentration in the lumen of the intestine, which triggers inflammatory signaling pathways in the intestine via toll-like receptor (TLR4) activation. Consequently, disturbances in the production, secretion and thickness of the mucus layer occur, promoting the passage of bacterial components from the intestinal lumen into the circulation and peripheral tissues, which ultimately leads to the development of systemic inflammation, adipogenesis, insulin resistance and hyperglycemia [68]. Another study has shown that low fiber intake contributes to the increase in the number of mucus-decomposing bacteria, including *Akmermansia muciniphila* and *Bacteroides caccae*. The mucus layer becomes thinner, which leads to higher susceptibility to pathogens inducing colitis [69].

Beside psychological distress and poor nutritional habits, the intake of the common, over the counter medicines, PPIs and NSAIDs, was found to be responsible for particular digestive complaints. However, such association with PPIs was lost, as presented by means of the FDR *p* value. Proton pump inhibitors intake was identified as factor escalating the intensity of leaky gut syndrome and gastric ulcers symptoms whilst NSAIDs shared these associations along with triggering the sensitivity of the stomach. NSAIDs are absorbed in enterocytes, which further inhibits the oxidative phosphorylation in the mitochondria and results in the destruction of TJs [70]. NSAIDs also inhibit prostaglandin-endoperoxide synthase 1 and 2 (COX1 and COX2) previously shown to be responsible for gastric and small intestine damage via altered secretion of gastric acid, mucus and bicarbonate as well as decreased epithelial cell proliferation and blood flow [71]. Indeed, beside cell viability, uninterrupted blood flow plays a fundamental role in the protection and healing of mucosa in the colon [72]. Ghrelin administered to animals with dextran sulfate sodium -induced colitis restored both the DNA synthesis, and normal blood flow contributing to the preventive effect against progression of DSS-induced colitis [73]. Similar results were obtained for obestatin in the case of aceticacid-induced colitis [74] and trinitrobenzene sulfonic acid (TNBS)-induced colitis [75]. PPIs were recognized as important dysbiotic medicines increasing susceptibility to *Clostridium difficile* infection [76] as well as small intestinal bacterial overgrowth, bacterial peritonitis, and poor outcomes in inflammatory bowel disease [77]. In a very recent study le Bastard et al. found that both PPIs, and NSAIDs increased the abundance of pathogenic *Enterobacter*, *Escherichia*, *Klebsiella* and *Citrobacter* or different genera of family *Enterococcaceae* [78].

Lastly, we found that women’s experience of environmental factors, possibly leading to microbiome alterations and digestive complaints, were more intense in comparison to men. The higher frequency of intestinal problems in women was previously observed [79] probably due to the menstrual cycle, with sex hormones modulating sensitivity to physical stress and perception of visceral hypersensitivity [80]. Socio-cultural factors discussed earlier may be of relevance as well [51]. However, this association need to be taken cautiously, as it was evaluated that in the USA almost all women of reproductive age with a history of sexual intercourse have used contraceptives in their lifetime [81]. In Poland contraceptive prevalence was found to be around 70% [82]. These issues may be the cause of potential bias of the results presented in our study.

## 5. Conclusions

In our present study we surveyed a large sample of representative inhabitants of Poland which is the key strength of the research. Our study however utilized a non-validated questionnaire with symptom overlaps thus its results must be cautiously analyzed. In conclusion, we found that gastrointestinal symptoms are common in the Polish population. In addition, psychological stress, unhealthy diets and over the counter medication usage may be responsible, at least partly, for these digestive complaints. Further attention should be focused on these environmental factors influencing the gut-brain axis and thus public health. Our results should be confirmed with other validated questionnaires (e.g., ROME IV criteria FGIDs questionnaire) and currently the effort to conduct new studies has been undertaken at our center.

## Figures and Tables

**Table 1:** Transformation of numerical data into descriptive data in the Digestive Health Appraisal Questionnaire (DHAQ).

Section	Number of Points Stating Priority for Healing Process			
	Low Priority	Moderate Priority	High Priority	
A	0–4	5–8	>9	
B	0–4	5–8	>9	
C	0–4	5–8	>9	
D	0–4	5–8	>9	
E	0–2	3–5	>6	
G	0–3	4–6	>7	
	Low priority	Mild priority	Moderate priority	High priority
F	0–5	6–10	7–19	>20

**Table 2:** Study group characteristics. Descriptive statistics of indices from the second part of the DHAQ are presented.

Variable	Mean	Standard Deviation	Median
Age (years)	25.20	6.32	24
Medications used currently	3.79	4.92	1
Food, nutrition	5.12	2.72	5
Lifestyle	1.36	1.00	1
Overall dysbiotic factors	10.27	5.75	10
Hypoacidity of the stomach	3.80	3.18	3
Hypofunction of small intestines and/or pancreas	6.40	5.62	5
Ulcers/hyperacidity of the stomach	3.10	4.01	2
Colon/large intestine	4.74	3.45	4
Liver/gallbladder	5.28	5.17	4
Intestinal permeability/leaky gut syndrome, dysbiosis	6.38	5.11	6
Gastric reflux	1.86	2.42	1

**Table 3:** The comparison of intensity of gastrointestinal symptoms in terms of gender by means of Mann-Whitney test.

Section	Median (Q3–Q1)		*p*
	Females	Males	
	(*n* = 239)	(*n* = 189)	
Hypoacidity of the stomach	4.0 (4.0)	3.0 (4.0)	0.0012
Hypofunction of small intestines and/or pancreas	6.0 (9.0)	5.0 (6.0)	0.017
Ulcers/hyperacidity of the stomach	3.0 (5.0)	1.0 (4.0)	<0.0001
Colon/large intestine	5.0 (5.0)	4.0 (2.0)	<0.0001
Liver/gallbladder	5.0 (8.0)	3.0 (5.25)	<0.0001
Intestinal permeability/leaky gut syndrome, dysbiosis	6.0 (7.0)	4.0 (6.0)	<0.0001
Gastric reflux	1.0 (3.0)	4.0 (6.0)	0.1857

**Table 4:** The comparison of priority of gastrointestinal symptoms in terms of gender by means of Chi-squared test. Number of subjects are presented.

Variable	Hypoacidity of the Stomach (*n* = 156) *			*p*	
Gender	Low Priority	Moderate Priority	High Priority		
	*n* (%)	*n* (%)	*n* (%)		
Females	143 (59.83)	64 (26.78)	32 (13.39)	0.0005	
Males	129 (68.25)	54 (28.57)	6 (3.17)		
	Hypofunction of small intestines and/or pancreas (*n* = 233) *			*p*	
Females	106 (44.35)	47 (19.66)	86 (35.98)	0.0153	
Males	89 (47.09)	54 (28.57)	46 (24.34)		
	Ulcers/hyperacidity of the stomach (*n* = 109) *			*p*	
Females	167 (69.87)	45 (18.83)	27 (11.30)	0.04	
Males	152 (80.42)	25 (13.23)	12 (6.35)		
	Colon/large intestine (*n* = 201) *			*p*	
Females	107 (44.77)	93 (38.91)	39 (16.32)	0.0001	
Males	120 (63.49)	57 (30.16)	12 (6.35)		
	Liver/gallbladder (*n* = 266) *			*p*	
Females	82 (34.31)	40 (16.74)	117 (48.95)	<0.0001	
Males	80 (42.33)	55 (29.10)	54 (28.57)		
	Gastric reflux (*n* = 83) *			*p*	
Females	192 (80.33)	31 (12.15)	16 (6.69)	0.2047	
Males	153 (80.95)	30 (15.87)	6 (3.17)		
Intestinal permeability/leaky gut syndrome, dysbiosis (*n* = 219) *					*p*
	Low priority	Mild priority	Moderate priority	High priority	
Females	93 (38.91)	84 (35.15)	55 (23.01)	7 (2.93)	<0.0001
Males	116 (61.37)	48 (25.40)	25 (25.40)	(0)	

* without low priority cases.

**Table 5:** The association between certain dysbiotic factors and the intensity of digestive complaints by means of Mann-Whitney test. Nonsteroidal anti-inflammatory drugs (NSAIDs); proton pump inhibitors (PPIs); false discovery rate (FDR).

Median (Q3–Q1)				
Variable	ANTIBIOTICS (*n* = 168)	NO ANTIBIOTICS (*n* = 260)	*p* value	FDR *p* value
Hypoacidity of the stomach	4.0 (5.0)	3.0 (4.0)	0.0808	0.2767
Hypofunction of small intestines and/or pancreas	6.0 (8.0)	5.0 (7.0)	0.3459	0.3459
Ulcers/hyperacidity of the stomach	2.0 (5.0)	2.0 (4.0)	0.2011	0.2767
Colon/large intestine	4.5 (4.0)	4.0 (4.0)	0.2372	0.2767
Liver/gallbladder	4.0 (6.5)	4.0 (6.5)	0.1755	0.2767
Intestinal permeability/leaky gut syndrome, dysbiosis	6.0 (7.0)	5.0 (7.0)	0.0704	0.2767
Gastric reflux	1.0 (3.0)	1.0 (3.0)	0.1857	0.2767
Variable	PPIs (*n* = 121)	NO PPIs (*n* = 307)	*p*	FDR *p* value
Hypoacidity of the stomach	3.0 (5.0)	3.0 (4.0)	0.2226	0.3116
Hypofunction of small intestines and/or pancreas	5.0 (8.25)	5.0 (7.0)	0.7345	0.7345
Ulcers/hyperacidity of the stomach	2.0 (6.0)	2.0 (4.0)	0.0902	0.1933
Colon/large intestine	4.0 (4.25)	1.0 (3.0)	0.3267	0.3812
Liver/gallbladder	4.0 (7.0)	4.0 (6.0)	0.1105	0.1934
Intestinal permeability/leaky gut syndrome, dysbiosis	6.0 (8.0)	5.0 (7.0)	0.0288	0.1484
Gastric reflux	1.0 (4.0)	1.0 (3.0)	0.0424	0.1484
Variable	NSAIDs (*n* = 182)	NO NSAIDs (*n* = 246)	*p*	FDR *p* value
Hypoacidity of the stomach	4.0 (5.0)	1.0 (3.0)	0.0101	0.0353
Hypofunction of small intestines and/or pancreas	6.0 (8.0)	4.5 (7.0)	0.2892	0.2892
Ulcers/hyperacidity of the stomach	2.0 (5.0)	1.0 (4.0)	0.0626	0.1095
Colon/large intestine	4.0 (4.0)	4.0 (4.0)	0.2614	0.2892
Liver/gallbladder	4.0 (7.0)	3.0 (6.0)	0.0184	0.0429
Intestinal permeability/leaky gut syndrome, dysbiosis	6.0 (7.0)	4.0 (7.0)	0.008	0.0353
Gastric reflux	1.0 (3.0)	1.0 (2.0)	0.0893	0.1250
Variable	Permanent stress (*n* = 149)	No permanent stress (*n* = 279)	*p*	FDR *p* value
Hypoacidity of the stomach	5.0 (5.0)	3.0 (4.0)	<0.0001	0.0001
Hypofunction of small intestines and/or pancreas	8.0 (10.0)	4.0 (6.0)	<0.0001	0.0001
Ulcers/hyperacidity of the stomach	4.0 (6.0)	1.0 (3.0)	<0.0001	0.0001
Colon/large intestine	6.0 (4.0)	3.0 (3.0)	<0.0001	0.0001
Liver/gallbladder	7.0 (8.0)	3.0 (5.0)	<0.0001	0.0001
Intestinal permeability/leaky gut syndrome, dysbiosis	9.0 (7.5)	5.0 (5.0)	<0.0001	0.0001
Gastric reflux	2.0 (4.0)	1.0 (2.0)	0.0008	0.0008

**Table 6:** Correlation between number of declared dysbiotics and intensity of gastrointestinal symptoms by means of Spearman’s rank correlation analysis.

Dysbiotic Agents vs. Intensity of:	Correlation	*p*	FDR *p*
Hypoacidity of the stomach	0.25	<0.0001	0.0001
Hypofunction of small intestines and/or pancreas	0.21	<0.0001	0.0001
Ulcers/hyperacidity of the stomach	0.24	<0.0001	0.0001
Colon/large intestine	0.25	<0.0001	0.0001
Liver/gallbladder	0.23	<0.0001	0.0001
Intestinal permeability/leaky gut syndrome, dysbiosis	0.27	<0.0001	0.0001
Gastric reflux	0.26	<0.0001	0.0001

**Table 7:** The association between the number of declared dysbiotics and gastrointestinal symptoms by means of the Kruskal-Wallis test.

Variable	Median (Q3–Q1)			*p*	FDR *p* Value
	Low Priority	Moderate Priority	High Priority		
Hypoacidity of the stomach	9.0 (8.0)	10. (8.0)	12.0 (7.0)	<0.0001	0.0001
Hypofunction of small intestines and/or pancreas	8.0 (9.0)	11.0 (7.0)	11.0 (9.0)	<0.0001	0.0001
Ulcers/hyperacidity of the stomach	9.0 (9.0)	10.0 (7.0)	15.0 (11.75)	<0.0001	0.0001
Colon/large intestine	9.0 (8.0)	10.0 (8.0)	12.0 (8.75)	<0.0001	0.0001
Liver/gallbladder	8.0 (8.0)	10.0 (7.0)	10.0 (9.75)	<0.0001	0.0001
Gastric reflux	9.0 (9.0)	10.0 (9.0)	11.5 (10.0)	<0.0001	0.0001
Intestinal permeability/leaky gut syndrome, dysbiosis	9.0 (9.0)	10. (9.0)	20.0 (8.5)	<0.0001	0.0001

## References

[1] Stanghellini V. (2017). Functional Dyspepsia and Irritable Bowel Syndrome: Beyond Rome IV. Digest. Dis..

[2] Francisconi C.F., Sperber A.D., Fang X., Fukudo S., Gerson M.J., Kang J.Y., Schmulson M. (2016). Multicultural Aspects in Functional Gastrointestinal Disorders (FGIDs). Gastroenterology.

[3] Robin S.G., Keller C., Zwiener R., Hyman P.E., Nurko S., Saps M., Di Lorenzo C., Shulman R.J., Hyams J.S., Palsson O. (2018). Prevalence of Pediatric Functional Gastrointestinal Disorders Utilizing the Rome IV Criteria. J. Pediatr..

[4] Tack J., Corsetti M., Camilleri M., Quigley E.M., Simren M., Suzuki H., Talley N.J., Tornblom H., van Oudenhove L. (2017). Plausibility criteria for putative pathophysiological mechanisms in functional gastrointestinal disorders: A consensus of experts. Gut.

[5] Drossman D.A. (2016). Functional Gastrointestinal Disorders: History, Pathophysiology, Clinical Features and Rome IV. Gastroenterology.

[6] Schmulson M.J., Drossman D.A. (2017). What Is New in Rome IV. J. Neurogastroenterol. Motil..

[7] Lynch S.V., Pedersen O. (2016). The Human Intestinal Microbiome in Health and Disease. N. Engl. J. Med..

[8] Li J., Jia H., Cai X., Zhong H., Feng Q., Sunagawa S., Arumugam M., Kultima J.R., Prifti E., Nielsen T. (2014). MetaHIT Consortium An integrated catalog of reference genes in the human gut microbiome. Nat. Biotechnol..

[9] Yatsunenko T., Rey F.E., Manary M.J., Trehan I., Dominguez-Bello M.G., Contreras M., Magris M., Hidalgo G., Baldassano R.N., Anokhin A.P. (2012). Human gut microbiome viewed across age and geography. Nature.

[10] Qin J., Li R., Raes J., Arumugam M., Burgdorf K.S., Manichanh C., Nielsen T., Pons N., Levenez F., Yamada T. (2010). A human gut microbial gene catalogue established by metagenomic sequencing. Nature.

[11] Consortium T.H.M.P., Huttenhower C., Gevers D., Knight R., Abubucker S., Badger J.H., Chinwalla A.T., Creasy H.H., Earl A.M., FitzGerald M.G. (2012). Structure, function and diversity of the healthy human microbiome. Nature.

[12] Sonnenburg J.L., Bäckhed F. (2016). Diet-microbiota interactions as moderators of human metabolism. Nature.

[13] Koppel N., Maini Rekdal V., Balskus E.P. (2017). Chemical transformation of xenobiotics by the human gut microbiota. Science.

[14] Kamada N., Chen G.Y., Inohara N., Núñez G. (2013). Control of pathogens and pathobionts by the gut microbiota. Nat. Immunol..

[15] Ijssennagger N., Belzer C., Hooiveld G.J., Dekker J., van Mil S.W.C., Müller M., Kleerebezem M., van der Meer R. (2015). Gut microbiota facilitates dietary heme-induced epithelial hyperproliferation by opening the mucus barrier in colon. Proc. Natl. Acad. Sci. USA.

[16] Blander J.M., Longman R.S., Iliev I.D., Sonnenberg G.F., Artis D. (2017). Regulation of inflammation by microbiota interactions with the host. Nat. Immunol..

[17] Fulde M., Hornef M.W. (2014). Maturation of the enteric mucosal innate immune system during the postnatal period. Immunol. Rev..

[18] Reinhardt C., Bergentall M., Greiner T.U., Schaffner F., Ostergren-Lundén G., Petersen L.C., Ruf W., Bäckhed F. (2012). Tissue factor and PAR1 promote microbiota-induced intestinal vascular remodelling. Nature.

[19] Novince C.M., Whittow C.R., Aartun J.D., Hathaway J.D., Poulides N., Chavez M.B., Steinkamp H.M., Kirkwood K.A., Huang E., Westwater C. (2017). Commensal Gut Microbiota Immunomodulatory Actions in Bone Marrow and Liver have Catabolic Effects on Skeletal Homeostasis in Health. Sci. Rep..

[20] Federico A., Dallio M., DI Sarno R., Giorgio V., Miele L. (2017). Gut microbiota, obesity and metabolic disorders. Minerva Gastroenterol. Dietol..

[21] Jandhyala S.M., Talukdar R., Subramanyam C., Vuyyuru H., Sasikala M., Nageshwar Reddy D. (2015). Role of the normal gut microbiota. World J. Gastroenterol..

[22] Dinan T.G., Cryan J.F., Stanton C. (2018). Gut Microbes and Brain Development Have Black Box Connectivity. Biol. Psychiatry.

[23] Vancamelbeke M., Vermeire S. (2017). The intestinal barrier: A fundamental role in health and disease. Expert Rev. Gastroenterol. Hepatol..

[24] Vanheel H., Vicario M., Vanuytsel T., Van Oudenhove L., Martinez C., Keita Å.V., Pardon N., Santos J., Söderholm J.D., Tack J. (2014). Impaired duodenal mucosal integrity and low-grade inflammation in functional dyspepsia. Gut.

[25] Barbara G., Feinle-Bisset C., Ghoshal U.C., Quigley E.M., Santos J., Vanner S., Vergnolle N., Zoetendal E.G. (2016). The Intestinal Microenvironment and Functional Gastrointestinal Disorders. Gastroenterology.

[26] Bischoff S.C., Barbara G., Buurman W., Ockhuizen T., Schulzke J.-D., Serino M., Tilg H., Watson A., Wells J.M. (2014). Intestinal permeability—A new target for disease prevention and therapy. BMC Gastroenterol..

[27] Barbara G., Wang B., Stanghellini V., de Giorgio R., Cremon C., Di Nardo G., Trevisani M., Campi B., Geppetti P., Tonini M. (2007). Mast cell-dependent excitation of visceral-nociceptive sensory neurons in irritable bowel syndrome. Gastroenterology.

[28] Dembiński A., Warzecha Z., Ceranowicz P., Konturek S.J. (1995). The role of capsaicin-sensitive sensory neurons and nitric oxide in regulation of gastric mucosal growth. J. Physiol. Pharmacol..

[29] Dembinski A., Warzecha Z., Konturek P.J., Ceranowicz P., Konturek S.J. (1996). Influence of capsaicin-sensitive afferent neurons and nitric oxide (NO) on cerulein-induced pancreatitis in rats. Int. J. Pancreatol..

[30] Warzecha Z., Dembiński A., Ceranowicz P., Stachura J., Tomaszewska R., Konturek S.J. (2001). Effect of sensory nerves and CGRP on the development of caerulein-induced pancreatitis and pancreatic recovery. J. Physiol. Pharmacol..

[31] Warzecha Z., Dembiński A., Ceranowicz P., Konturek P.C., Stachura J., Tomaszewska R., Konturek S.J. (1999). Calcitonin gene-related peptide can attenuate or augment pancreatic damage in caerulein-induced pancreatitis in rats. J. Physiol. Pharmacol..

[32] Holzer P., Farzi A. (2014). Neuropeptides and the Microbiota-Gut-Brain Axis. Adv. Exp. Med. Biol..

[33] Pimentel M., Lin H.C., Enayati P., van den Burg B., Lee H.-R., Chen J.H., Park S., Kong Y., Conklin J. (2006). Methane, a gas produced by enteric bacteria, slows intestinal transit and augments small intestinal contractile activity. Am. J. Physiol. Gastrointest. Liver Physiol..

[34] Carbonero F., Benefiel A.C., Gaskins H.R. (2012). Contributions of the microbial hydrogen economy to colonic homeostasis. Nat. Rev. Gastroenterol. Hepatol..

[35] Teague B., Asiedu S., Moore P.K. (2002). The smooth muscle relaxant effect of hydrogen sulphide in vitro: Evidence for a physiological role to control intestinal contractility. Br. J. Pharmacol..

[36] McCusker R.H., Kelley K.W. (2013). Immune-neural connections: How the immune system’s response to infectious agents influences behavior. J. Exp. Biol..

[37] Hoyles L., Snelling T., Umlai U.-K., Nicholson J.K., Carding S.R., Glen R.C., McArthur S. (2018). Microbiome-host systems interactions: Protective effects of propionate upon the blood-brain barrier. Microbiome.

[38] Mayer E. (2000). The neurobiology of stress and gastrointestinal disease. Gut.

[39] Stasi C., Nisita C., Cortopassi S., Corretti G., Gambaccini D., De Bortoli N., Fani B., Simonetti N., Ricchiuti A., Dell’Osso L. (2017). Subthreshold Psychiatric Psychopathology in Functional Gastrointestinal Disorders: Can It Be the Bridge between Gastroenterology and Psychiatry?. Gastroenterol. Res. Pract..

[40] Niemyjska S., Ukleja A., Ławiński M. (2015). Evaluation of Irritable Bowel Syndrome Symptoms Amongst Warsaw University Students. Pol. Przegl. Chir..

[41] Ziółkowski B.A., Pacholec A., Kudlicka M., Ehrmann A., Muszyński J. (2012). Prevalence of abdominal symptoms in the Polish population. Gastroenterol. Rev..

[42] Maciejewska D., Michalczyk M., Czerwińska-Rogowska M., Banaszczak M., Ryterska K., Jakubczyk K., Piotrwski J., Hołowko J., Drozd A., Wysokińki P. (2017). Seeking Optimal Nutrition for Healthy Body Mass Reduction among Former Athletes. J. Hum. Kinet..

[43] Digestive Wellness. https://www.goodreads.com/work/best_book/1264033-digestive-wellness.

[44] Drossman D.A., Camilleri M., Mayer E.A., Whitehead W.E. (2002). AGA technical review on irritable bowel syndrome. Gastroenterology.

[45] Lacy B.E., Patel N.K. (2017). Rome Criteria and a Diagnostic Approach to Irritable Bowel Syndrome. J. Clin. Med..

[46] Aziz I., Palsson O.S., Törnblom H., Sperber A.D., Whitehead W.E., Simrén M. (2018). Epidemiology, clinical characteristics, and associations for symptom-based Rome IV functional dyspepsia in adults in the USA, Canada, and the UK: A cross-sectional population-based study. Lancet Gastroenterol. Hepatol..

[47] Aziz I., Palsson O.S., Törnblom H., Sperber A.D., Whitehead W.E., Simrén M. (2018). The Prevalence and Impact of Overlapping Rome IV-Diagnosed Functional Gastrointestinal Disorders on Somatization, Quality of Life, and Healthcare Utilization: A Cross-Sectional General Population Study in Three Countries. Am. J. Gastroenterol..

[48] Sperber A.D., Dumitrascu D., Fukudo S., Gerson C., Ghoshal U.C., Gwee K.A., Hungin A.P.S., Kang J.-Y., Minhu C., Schmulson M. (2017). The global prevalence of IBS in adults remains elusive due to the heterogeneity of studies: A Rome Foundation working team literature review. Gut.

[49] Holtmann G.J., Talley N.J. (2018). Inconsistent symptom clusters for functional gastrointestinal disorders in Asia: Is Rome burning?. Gut.

[50] Palsson O.S., Whitehead W.E., van Tilburg M.A.L., Chang L., Chey W., Crowell M.D., Keefer L., Lembo A.J., Parkman H.P., Rao S.S. (2016). Rome IV Diagnostic Questionnaires and Tables for Investigators and Clinicians. Gastroenterology.

[51] Rahman M.M., Mahadeva S., Ghoshal U.C. (2017). Epidemiological and clinical perspectives on irritable bowel syndrome in India, Bangladesh and Malaysia: A review. World J. Gastroenterol..

[52] Zhong L., Shanahan E.R., Raj A., Koloski N.A., Fletcher L., Morrison M., Walker M.M., Talley N.J., Holtmann G. (2017). Dyspepsia and the microbiome: Time to focus on the small intestine. Gut.

[53] Talley N.J. (2017). Editorial: Moving Away from Focussing on Gastric Pathophysiology in Functional Dyspepsia: New Insights and Therapeutic Implications. Am. J. Gastroenterol..

[54] Malinen E., Rinttilä T., Kajander K., Mättö J., Kassinen A., Krogius L., Saarela M., Korpela R., Palva A. (2005). Analysis of the fecal microbiota of irritable bowel syndrome patients and healthy controls with real-time PCR. Am. J. Gastroenterol..

[55] Rajilić-Stojanović M., Biagi E., Heilig H.G.H.J., Kajander K., Kekkonen R.A., Tims S., de Vos W.M. (2011). Global and deep molecular analysis of microbiota signatures in fecal samples from patients with irritable bowel syndrome. Gastroenterology.

[56] Giamarellos-Bourboulis E., Tang J., Pyleris E., Pistiki A., Barbatzas C., Brown J., Lee C.C., Harkins T.T., Kim G., Weitsman S. (2015). Molecular assessment of differences in the duodenal microbiome in subjects with irritable bowel syndrome. Scand. J. Gastroenterol..

[57] Vicario M., González-Castro A.M., Martínez C., Lobo B., Pigrau M., Guilarte M., de Torres I., Mosquera J.L., Fortea M., Sevillano-Aguilera C. (2015). Increased humoral immunity in the jejunum of diarrhoea-predominant irritable bowel syndrome associated with clinical manifestations. Gut.

[58] Park J.H., Rhee P.-L., Kim H.S., Lee J.H., Kim Y.-H., Kim J.J., Rhee J.C. (2006). Mucosal mast cell counts correlate with visceral hypersensitivity in patients with diarrhea predominant irritable bowel syndrome. J. Gastroenterol. Hepatol..

[59] Martínez C., Vicario M., Ramos L., Lobo B., Mosquera J.L., Alonso C., Sánchez A., Guilarte M., Antolín M., de Torres I. (2012). The jejunum of diarrhea-predominant irritable bowel syndrome shows molecular alterations in the tight junction signaling pathway that are associated with mucosal pathobiology and clinical manifestations. Am. J. Gastroenterol..

[60] Rothschild D., Weissbrod O., Barkan E., Kurilshikov A., Korem T., Zeevi D., Costea P.I., Godneva A., Kalka I.N., Bar N. (2018). Environment dominates over host genetics in shaping human gut microbiota. Nature.

[61] Foster J.A., Rinaman L., Cryan J.F. (2017). Stress & the gut-brain axis: Regulation by the microbiome. Neurobiol. Stress.

[62] Koloski N.A., Jones M., Talley N.J. (2016). Evidence that independent gut-to-brain and brain-to-gut pathways operate in the irritable bowel syndrome and functional dyspepsia: A 1-year population-based prospective study. Aliment. Pharmacol. Ther..

[63] Jones M.P., Tack J., Van Oudenhove L., Walker M.M., Holtmann G., Koloski N.A., Talley N.J. (2017). Mood and Anxiety Disorders Precede Development of Functional Gastrointestinal Disorders in Patients but Not in the Population. Clin. Gastroenterol. Hepatol..

[64] Qin H.-Y., Cheng C.-W., Tang X.-D., Bian Z.-X. (2014). Impact of psychological stress on irritable bowel syndrome. World J. Gastroenterol..

[65] Konturek P.C., Brzozowski T., Konturek S.J. (2011). Stress and the gut: Pathophysiology, clinical consequences, diagnostic approach and treatment options. J. Physiol. Pharmacol..

[66] Rodiño-Janeiro B.K., Alonso-Cotoner C., Pigrau M., Lobo B., Vicario M., Santos J. (2015). Role of Corticotropin-releasing Factor in Gastrointestinal Permeability. J. Neurogastroenterol. Motil..

[67] Singh R.K., Chang H.-W., Yan D., Lee K.M., Ucmak D., Wong K., Abrouk M., Farahnik B., Nakamura M., Zhu T.H. (2017). Influence of diet on the gut microbiome and implications for human health. J. Transl. Med..

[68] Araújo J.R., Tomas J., Brenner C., Sansonetti P.J. (2017). Impact of high-fat diet on the intestinal microbiota and small intestinal physiology before and after the onset of obesity. Biochimie.

[69] Desai M.S., Seekatz A.M., Koropatkin N.M., Kamada N., Hickey C.A., Wolter M., Pudlo N.A., Kitamoto S., Terrapon N., Muller A. (2016). A Dietary Fiber-Deprived Gut Microbiota Degrades the Colonic Mucus Barrier and Enhances Pathogen Susceptibility. Cell.

[70] Matsui H., Shimokawa O., Kaneko T., Nagano Y., Rai K., Hyodo I. (2011). The pathophysiology of non-steroidal anti-inflammatory drug (NSAID)-induced mucosal injuries in stomach and small intestine. J. Clin. Biochem. Nutr..

[71] Bjarnason I., Scarpignato C., Holmgren E., Olszewski M., Rainsford K.D., Lanas A. (2018). Mechanisms of Damage to the Gastrointestinal Tract From Nonsteroidal Anti-Inflammatory Drugs. Gastroenterology.

[72] Leung F.W., Su K.C., Pique J.M., Thiefin G., Passaro E., Guth P.H. (1992). Superior mesenteric artery is more important than inferior mesenteric artery in maintaining colonic mucosal perfusion and integrity in rats. Digest. Dis. Sci..

[73] Matuszyk A., Ceranowicz D., Warzecha Z., Ceranowicz P., Fyderek K., Gałązka K., Cieszkowski J., Bonior J., Jaworek J., Pihut M. (2015). The Influence of Ghrelin on the Development of Dextran Sodium Sulfate-Induced Colitis in Rats., The Influence of Ghrelin on the Development of Dextran Sodium Sulfate-Induced Colitis in Rats. Biomed. Res. Int..

[74] Matuszyk A., Ceranowicz P., Warzecha Z., Cieszkowski J., Bonior J., Jaworek J., Kuśnierz-Cabala B., Konturek P., Ambroży T., Dembiński A. (2016). Obestatin Accelerates the Healing of Acetic Acid-Induced Colitis in Rats. Oxid. Med. Cell. Longev..

[75] Konarska K., Cieszkowski J., Warzecha Z., Ceranowicz P., Chmura A., Kuśnierz-Cabala B., Gałązka K., Kowalczyk P., Miskiewicz A., Konturek T.J. (2018). Treatment with Obestatin-A Ghrelin Gene-Encoded Peptide-Reduces the Severity of Experimental Colitis Evoked by Trinitrobenzene Sulfonic Acid. Int. J. Mol. Sci..

[76] Takagi T., Naito Y., Inoue R., Kashiwagi S., Uchiyama K., Mizushima K., Tsuchiya S., Okayama T., Dohi O., Yoshida N. (2018). The influence of long-term use of proton pump inhibitors on the gut microbiota: An age-sex-matched case-control study. J. Clin. Biochem. Nutr..

[77] Naito Y., Kashiwagi K., Takagi T., Andoh A., Inoue R. (2018). Intestinal Dysbiosis Secondary to Proton-Pump Inhibitor Use. Digestion.

[78] Le Bastard Q., Al-Ghalith G.A., Grégoire M., Chapelet G., Javaudin F., Dailly E., Batard E., Knights D., Montassier E. (2018). Systematic review: Human gut dysbiosis induced by non-antibiotic prescription medications. Aliment. Pharmacol. Ther..

[79] Adeyemo M.A., Spiegel B.M.R., Chang L. (2010). Meta-analysis: Do irritable bowel syndrome symptoms vary between men and women?. Aliment. Pharmacol. Ther..

[80] Meleine M., Matricon J. (2014). Gender-related differences in irritable bowel syndrome: Potential mechanisms of sex hormones. World J. Gastroenterol..

[81] Daniels K., Mosher W.D. (2013). Contraceptive Methods Women Have ever Used: United States, 1982–2010.

[82] Family Planning—United Nations Population Division | Department of Economic and Social Affairs.. http://www.un.org/en/development/desa/population/publications/dataset/contraception/wcu2017.shtml.

